# The Finite Element Method Applied to a Problem of Blood Flow in Vessels

**DOI:** 10.1155/2012/204926

**Published:** 2012-01-26

**Authors:** Gabriela Nuţ, Ioana Chiorean, Maria Crişan

**Affiliations:** ^1^Faculty of Mathematics and Computer Science, Babeş-Bolyai University 1, Mihail Kogălniceanu Street, 400084 Cluj Napoca, Romania; ^2^Clinic of Dermatology, Department of Histology, Iuliu Hatieganu University of Medicine and Pharmacy 8 Victor Babeş Street, 400012 Cluj-Napoca, Romania

## Abstract

We use the finite element method to solve a convection-diffusion equation when convection is dominating, a problem which describes the behavior of the concentration of a solute in a blood vessel. A new technique for computing the discrete problem is used.

## 1. Introduction

Due to the fact that various chemicals, such as oxygen, carbon dioxide, or lipids, are transported by blood to and from the other tissues, including the skin, the study of the blood flow in the human vascular system is of great interest in medicine and medical engineering.

The skin is the largest organ of the human body, having very important psychosocial implications. It represents the only system completely displayed at the body surface, offering essential information regarding the homeostasis of the internal organs and thus the general senescence process. Senescence involves a complex of factors, of which vascularisation plays an important role. The perfusion degree of the tissues depends on the microscopical structure of the vascular walls, as well as the metabolical and biochemical changes associated with age. It is well known that the collagen glycation processes that occur in the vascular walls, and are involved in the senescence process, are associated with vascularisation deficits, that are specific to the ageing process generally, and to the associated pathology specifically (atherosclerosis, Alzheimer, metabolical diseases, rheumatoid arthritis). These modifications can be observed at the skin level by means of images techniques (see [1, 2]).

These are only few reasons for which the study of the possible solutes in blood vessels is so important. In this sense, many studies were made and different mathematical models were given, depending on various factors, such as the health state of the patient (the existence of pathologies like atherosclerosis, etc.). For instance, in [3], an implementation of adaptive anisotropic meshes for this class of problems by developing an a posteriori error analysis for a simpler situation, namely, a single steady advection-diffusion-reaction equation with a given convective field, is given.

## 2. The Model Problem

In order to study the transport problem of a solute in a vessel, we consider the following partial derivative equation:

(1) $$\begin{array}{cc} m\Delta c+n\nabla c & =f,\;\left(x,y\right)\in \Omega ,\\ c & =g_{1},\;\left(x,y\right)\in \Gamma_{\text{in}},\\ c & =g_{2},\;\left(x,y\right)\in \Gamma_{\text{out}},\\ c & =\xi g_{3},\;\left(x,y\right)\in \Gamma . \end{array}\;n=\left(n_{1},n_{2}\right).$$

Here, *c* = *c*(*x*, *y*) represents the concentration of the solute in the blood. The first term in (1) describes the diffusion, the later contains two more terms: one for the diffusion along the *x*-direction: *n*_1_(∂*c*/∂*x*), and another for the diffusion along the *y*-direction: *n*_2_(∂*c*/∂*y*). *m* represents the diffusivity of the solute, **n** a given velocity field, *α* is a reaction coefficient, *f*—a possible forcing term for the solution concentration due, for example, to chemical reactions.

The ratio between coefficients *m* and **n** determines the predominance of the convection or the diffusion in the physical process. If:

(2) $$\frac{\left|n\right|}{m}\gg 1,$$

then the convection is dominant. computing the numerical solution of a convection-diffusion problem of the form (1) becomes increasingly difficult (converge slow or not at all) as the ratio (2) increases (i.e., convection is dominant in the process), and this is the case in the model problem (1) (the ratio here is 10^4^, see Section 5).

**Figure 1:**
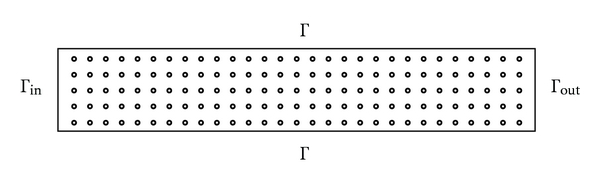


The domain *Ω* chosen for this problem represents a part of an artery (considered to be rigid) and is shown in Figure 1.

The borders of *Ω* are as follows.

- Γ_in_, which corresponds to the point in where the solution is injected in the blood, thus the concentration of the solution is known here: *u* = *g*_1_, (*x*, *y*) ∈ Γ_in_.
- Γ_out_, which corresponds to the point where the concentration of the solution can be measured: *u* = *g*_2_, (*x*, *y*) ∈ Γ_out_.
- Γ: in these points the solution is in contact with the vessel walls, *ξ* represents the wall permeability, and *g*_3_ is a function that decreases from *g*_1_ to *g*_2_.

## 3. The Discretization of the Problem

In this paper, we use the finite element method (see [4, 5]). In order to do this, as in [6–8], the domain [*a*, *b*]×[*c*, *d*] is divided in rectangular subdomains.

For the discretizaton of the problem we use the finite element method. The domain is divided in rectangular subdomains, having the step *h*_*x*_ on the *Ox*-direction and *h*_*y*_ on *Oy*. The solution of the systems generated through discretization is obtained by Gauss full elimination method. The first level on which the solution is computed is *l*_0_, then this particular solution is used for obtaining the solution on higher order levels. The grid on the *l* level is divided by the one from the *l*_0_ level in subdomains. On each of these, the corresponding system of linear equations will be solved, and the solutions from the subdomains are used in order to generate the solution on the *l* level.

The partial differential equations will be replaced by a liniar system of equations through the discretization method.

In order to achieve this, and keeping the notations used in [7], we choose the grid steps *h*_*lx*_ = (*b* − *a*)/2^*l*+1^ and *h*_*ly*_ = (*d* − *c*)/2^*l*+1^, *l* being the number of the level. The corresponding number of interior grid points is *n*_*l*_ = 2^*l*+1^ − 1 on each direction. The largest possible step corresponds to the level denoted by *l* = 0 on which the grid has a single point: ((*b* − *a*)/2, (*d* − *c*)/2). The grid on the level *l* will contain the points (*x*_*i*_, *y*_*j*_),   *i*, *j* = 1,2,…, *n*_*l*_, and will be denoted by *G*_*l*_. The value of the exact solution in the point (*x*_*i*_, *y*_*j*_) is denoted by c_*i*,*j*_.

### 3.1. Finite Element Discretization

According to [4], in order to apply the finite element discretization, some transformations of the given equation have to be made. So, the equation to be discretized is multiplied by a test function *v* ∈ *U*(*Ω*) = {*v* | *v* ∈ *H* ^1^(*Ω*), *v* = *g* on Γ}, then is integrated on the domain *Ω*; *H* ^1^(*Ω*) ⊂ *C* ^0^ is the space of functions with square integrable derivatives on *Ω*,

(3) $$\begin{array}{cc} & -m\iint_{\Omega }^{}\Delta cv\;dx\;dy+\iint_{\Omega }^{}n\nabla cv\;dx\;dy\\ & \;\;+\alpha \iint_{\Omega }^{}cv\;dx\;dy=\iint_{\Omega }^{}fv\;dx\;dy. \end{array}$$

Using Green's formula, the equation above becomes as follows:

(4) $$\begin{array}{cc} & m\iint_{\Omega }\nabla c\nabla v\;dx\;dy-m\int_{\delta \Omega }\frac{\partial c}{\partial n}\;v\;ds\\ & \;\;\;+\iint_{\Omega }n\nabla cv\;dx\;dy+\alpha \iint_{\Omega }cv\;dx\;dy\\ & \;\;=\iint_{\Omega }fv\;dx\;dy,\;u,\;\;v\in U\left(\Omega \right). \end{array}$$

The functions *c* and *v* are approximated using some continuous functions, Φ_*i*_ (Φ_*i*_(*x*_*j*_, *y*_*j*_) = *δ*_*ij*_,   *i*, *j* = 1,…, *N*, *N* = *n*_*l*_^2^ being the number of interior points of the grid on level *l*), through the following relations:

(5) $$c\approx \overset{N}{\underset{i=1}{\sum }}u_{i}\Phi_{i},\;\;v\approx \overset{N}{\underset{j=1}{\sum }}v_{j}\Phi_{j},$$

where *c*_*i*_ = *c*(*x*_*i*_, *y*_*i*_), *i* = 1,…, *N*. Replacing these approximations in (4), the system obtained is:

(6) $$\overset{N}{\underset{j=1}{\sum }}K_{ij}c_{j}\begin{array}{c} =F_{i},\;i=1,\ldots ,N \end{array},$$

where

(7) $$\begin{array}{cc} K_{ij} & =\iint_{\Omega }^{}[m\left(\frac{\partial \Phi_{i}}{\partial x}\frac{\partial \Phi_{j}}{\partial x}+\frac{\partial \Phi_{i}}{\partial y}\frac{\partial \Phi_{j}}{\partial y}\right)\\ & \;\;\;+n_{1}\Phi_{i}\frac{\partial \Phi_{j}}{\partial x}+n_{2}\Phi_{i}\frac{\partial \Phi_{j}}{\partial y}+\alpha \Phi_{i}\Phi_{j}]dx\;dy,\\ F_{i} & =\iint_{\Omega }^{}f\Phi_{i}\;dx\;dy. \end{array}$$

The restrictions of *K* and *F* on a domain *Ω*_*A*_ = [*a*, *b*]×[*c*, *d*] are as follows:

(8) $$\begin{array}{cc} k_{ij}^{A} & =m\iint_{\Omega_{A}}^{}\left(\frac{\partial \Psi_{i}^{A}}{\partial x}\frac{\partial \Psi_{j}^{A}}{\partial x}+\frac{\partial \Psi_{i}^{A}}{\partial y}\frac{\partial \Psi_{j}^{A}}{\partial y}\right)dx\;dy\\ & \;+n_{1}\iint_{\Omega_{A}}^{}\Psi_{i}^{A}\frac{\partial \Psi_{j}^{A}}{\partial x}dx\;dy+n_{2}\iint_{\Omega_{A}}^{}\Psi_{i}^{A}\frac{\partial \Psi_{j}^{A}}{\partial y}dx\;dy\\ & \;+\alpha \iint_{\Omega_{A}}^{}\Psi_{i}^{A}\Psi_{j}^{A}dx\;dy,\;i,j=1,\ldots ,4, \end{array}$$

(9) $$f_{k}^{A}=\iint_{\Omega_{A}}^{}f\left(x,y\right)\Psi_{k}^{A}\left(x,y\right)dx\;dy,\;k=1,\ldots ,4.$$

If *x* = (*d* − *c*)/(*b* − *a*), the values of the integral (8) for the problem (1) are given in the following 4 × 4 matrix:

(10) $$\begin{array}{cc} k & ={\left(k_{ij}\right)}_{i,j=\bar{1\;:\;4}}\\ & =\frac{m}{6}\left[\begin{array}{cccc} \;2x+\frac{2}{x} & -2x+\frac{1}{x} & -x-\frac{1}{x} & \;x-\frac{2}{x}\\ -2x+\frac{1}{x} & \;2x+\frac{2}{x} & \;x-\frac{2}{x} & -x-\frac{1}{x}\\ -x\;-\frac{1}{x} & \;x\;-\frac{2}{x} & \;2x+\frac{2}{x} & -2x+\frac{1}{x}\\ \;x\;-\frac{2}{x} & -x\;-\frac{1}{x} & -2x+\frac{1}{x} & \;2x+\frac{2}{x} \end{array}\right]\\ & \;+n_{1}\frac{d-c}{12}\left[\begin{array}{cccc} -2 & 2 & 1 & -1\\ -2 & 2 & 1 & -1\\ -1 & 1 & 2 & -2\\ -1 & 1 & 2 & -2 \end{array}\right]+n_{2}\frac{b-a}{12}\left[\begin{array}{cccc} -2 & -1 & 1 & 2\\ -1 & -2 & 2 & 1\\ -1 & -2 & 2 & 1\\ -2 & -1 & 1 & 2 \end{array}\right]\\ & \;+\alpha \frac{\left(b-a\right)\left(d-c\right)}{36}\left[\begin{array}{cccc} 4 & 2 & 1 & 2\\ 2 & 4 & 2 & 1\\ 1 & 2 & 4 & 2\\ 2 & 1 & 2 & 4 \end{array}\right]. \end{array}$$

Thus the differential equation (1) is approximated in a grid point (*x*_*i*_, *y*_*j*_),   *i*, *j* = 1,…, *n*_*l*_ by the following system of linear equations (see [9]):

(11) $$\begin{array}{cc} & \left[\left[\begin{array}{ccc} k_{24}^{D} & k_{23}^{D}+k_{14}^{C} & k_{13}^{C}\\ k_{34}^{A}+k_{21}^{D} & k_{33}^{A}+k_{44}^{B}+k_{11}^{C}+k_{22}^{D} & k_{43}^{B}+k_{12}^{C}\\ k_{31}^{A} & k_{32}^{A}+k_{41}^{B} & k_{42}^{B} \end{array}\right]\right]c_{i,j}\\ & \;\;=f_{3}^{A}+f_{4}^{B}+f_{1}^{C}+f_{2}^{D}, \end{array}$$

where

(12) $$\begin{array}{cc} \left[\left[\begin{array}{ccc} a & b & c\\ d & e & f\\ g & h & k \end{array}\right]\right]u_{i,j} & =au_{i-1,j+1}+bu_{i,j+1}+cu_{i+1,j+1}+du_{i-1,j}\\ & \;+eu_{i,j}+fu_{i+1,j}+gu_{i-1,j-1}+hu_{i,j-1}\\ & \;+ku_{i+1,j-1}. \end{array}$$

## 4. Solving Method

The systems generated in Section 3 can be written on any level *l*. Each system contains *n*_*l*_^2^ unknowns. The solution is exactly computed on a level *l*_0_, for example, on *l*_0_ = 2 or *l*_0_ = 3 using Gauss elimination method.

**Figure 2:**
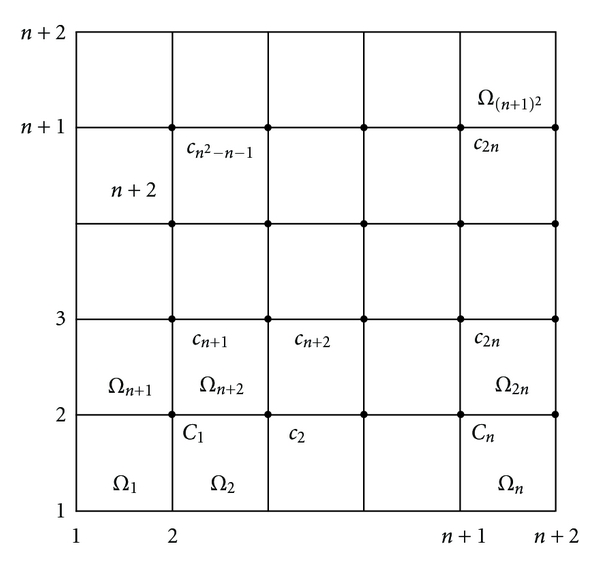


The exact solution on the level *l*_0_, for the problem is approximated by *c*_*i*_, *i* ∈ {1,2,…, *n*_*l*_0__^2^} (Figure 2), wich only contains an error term due to the discretization. In order to solve problem on the level *l*, the grid already obtained will be further divided. Thus, each domain from the grid, *Ω*_*k*_, will be splitted into *n*_*i*_ subdomains, where *n*_*i*_ = 2^*l*_*i*_+1^ − 1 and *l*_*i*_ = *l* − *l*_0_ − 1.

On each subdomain *Ω*_*k*_, the discretization of the differential equation leads to a system whose matrix has the same form as the one on *l*_0_ level. But on the level *l*_0_ the boundary values were given in the hypothesis. For the systems on the level *l* to be precisely solved on *Ω*_*k*_, one has to determine as accurate as possible the *n*_*i*_ values on each of the sides of the domain *Ω*_*k*_. Two possible ways to accomplish this are given in the following subsections.

### 4.1. Pondered Arithmetic Mean Prolongation

As in [7], the value of the approximation on level *l* is denoted by *c* ^(*l*)^. On the borders of *Ω*_*k*_, these values are defined through the following relations:

(13) $$\begin{array}{cc} c_{jN+1,iN+1}^{\left(l\right)} & =c_{(i-1)n+j}^{\left(l_{0}\right)},\\ c_{jN+1,iN+1+k}^{\left(l\right)} & =\frac{1}{N}\left(kc_{in+j}^{\left(l_{0}\right)}+\left(N-k\right)c_{(i-1)n+j}^{\left(l_{0}\right)}\right),\\ & \;i=0,\ldots ,n,\;j=1,\ldots ,n,\;N=n_{i}+1;\\ c_{jN+1+k,iN+1}^{\left(l\right)} & =\frac{1}{N}\left(kc_{(i-1)n+j+1}^{\left(l_{0}\right)}+\left(N-k\right)c_{(i-1)n+j}^{\left(l_{0}\right)}\right),\\ & \;i=1,\ldots ,n,\;j=0,\ldots ,n,\;k=1,\ldots ,n_{i}. \end{array}$$

### 4.2. Stellar Prolongation

In [9], a new type of prolongation which we called “stellar prolongation” because the nodes involved in computation are in the shape of a star, is presented. We use this technique in what follows, too.

**Figure 3:**
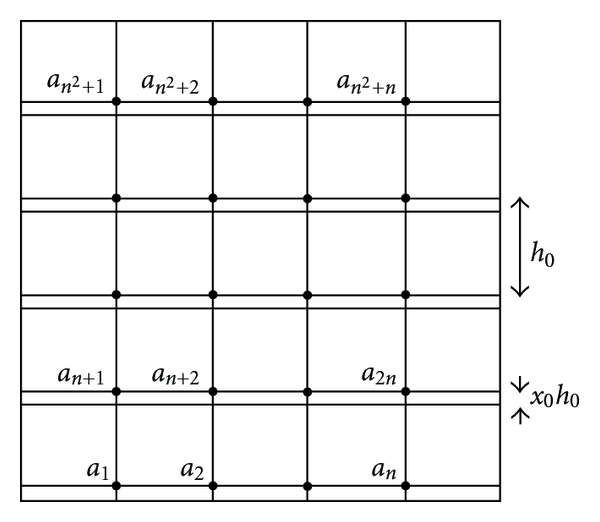


**Figure 4:**
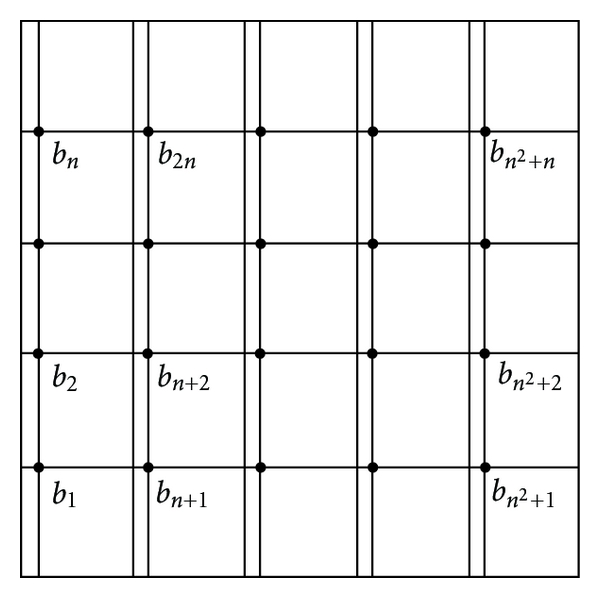


In order to determine more accurately the values of the solution on the borders of *Ω*_*k*_, instead of pondered arithmetic mean prolongation one can use the solutions of the systems obtained by discretizing the initial equation in the grid points corresponding to the values *a*_*i*_ and *b*_*i*_, *i* = 1,2,…, *n* ^2^ + *n* from Figures 3 and 4.

The values *a*_*k*_, *k* = 1,2,…, *n*(*n* + 1) are computed by solving a system with the following matrix:

(14) $$A=\left[\begin{array}{ccccccc} C & D & \Theta & \cdots & \Theta & \Theta & \\ S & C & D & \cdots & \Theta & \Theta & \\ \Theta & S & C & \cdots & \Theta & \Theta & \\ \vdots & & & \ddots & & & \\ \Theta & \Theta & \Theta & \cdots & S & C & \end{array}\right].$$

If the discretization is made by the finite element method, using the notation:

(15) $$\begin{array}{cc} & \left[\left[\begin{array}{ccc} l_{1} & l_{2} & l_{3}\\ l_{4} & l_{5} & l_{6}\\ l_{7} & l_{8} & l_{9} \end{array}\right]\right]\\ & =\left[\left[\begin{array}{ccc} k_{24}^{D} & k_{23}^{D}+k_{14}^{C} & k_{13}^{C}\\ k_{34}^{A}+k_{21}^{D} & k_{33}^{A}+k_{44}^{B}+k_{11}^{C}+k_{22}^{D} & k_{43}^{B}+k_{12}^{C}\\ k_{31}^{A} & k_{32}^{A}+k_{41}^{B} & k_{42}^{B} \end{array}\right]\right], \end{array}$$

where *k*_*ij*_ is given by (10), the matrix *A* has:

(16) $$\begin{array}{cc} C & =\left[\begin{array}{ccccc} l_{5} & l_{6} & 0 & \cdots & 0\\ l_{4} & l_{5} & l_{6} & \cdots & 0\\ 0 & l_{4} & l_{5} & \cdots & 0\\ \vdots & & & \ddots & \\ 0 & 0 & 0 & \cdots & l_{5} \end{array}\right],\\ D & =\left[\begin{array}{ccccc} l_{2} & l_{3} & 0 & \cdots & 0\\ l_{1} & l_{2} & l_{3} & \cdots & 0\\ 0 & l_{1} & l_{2} & \cdots & 0\\ \vdots & & & \ddots & \\ 0 & 0 & 0 & \cdots & l_{2} \end{array}\right],\\ S & =\left[\begin{array}{ccccc} l_{8} & l_{9} & 0 & \cdots & 0\\ l_{7} & l_{8} & l_{9} & \cdots & 0\\ 0 & l_{7} & l_{8} & \cdots & 0\\ \vdots & & & \ddots & \\ 0 & 0 & 0 & \cdots & l_{8} \end{array}\right]. \end{array}$$

The components of the constant terms vector are in this case:

(17) $$\begin{array}{cc} z_{in+j} & =f_{3}^{A}+f_{4}^{B}+f_{1}^{C}+f^{D}\\ & \;-\left[\left[\begin{array}{ccc} l_{1} & l_{2} & l_{3}\\ l_{4} & l_{5} & l_{6}\\ l_{7} & l_{8} & l_{9} \end{array}\right]\right]c_{fr}\left(jh,\left(i+x_{0}\right)h\right),\\ & \;\;\;i=0,\ldots ,n,\;j=1,\ldots ,n. \end{array}$$

*c*_*fr*_ is a function which is zero inside the domain *Ω* on wich the system is solved and is equal to the border values on *δΩ*, and *h* is the grid step on *l*_0_ level.

The values *b*_*k*_, *k* = 1,2,…, *n*(*n* + 1) are obtained from a system whose matrix is also of the form (14), but in which:

(18) $$\begin{array}{cc} & C=\left[\begin{array}{ccccc} l_{5} & l_{2} & 0 & \cdots & 0\\ l_{8} & l_{5} & l_{2} & \cdots & 0\\ 0 & l_{8} & l_{5} & \cdots & 0\\ \vdots & & & \ddots & \\ 0 & 0 & 0 & \cdots & l_{5} \end{array}\right],\\ & D=\left[\begin{array}{ccccc} l_{6} & l_{3} & 0 & \cdots & 0\\ l_{9} & l_{6} & l_{3} & \cdots & 0\\ 0 & l_{9} & l_{6} & \cdots & 0\\ \vdots & & & \ddots & \\ 0 & 0 & 0 & \cdots & l_{6} \end{array}\right],\\ & S=\left[\begin{array}{ccccc} l_{4} & l_{1} & 0 & \cdots & 0\\ l_{7} & l_{4} & l_{1} & \cdots & 0\\ 0 & l_{7} & l_{4} & \cdots & 0\\ \vdots & & & \ddots & \\ 0 & 0 & 0 & \cdots & l_{4} \end{array}\right]. \end{array}$$

The components of the constant terms vector are now

(19) $$\begin{array}{cc} z_{in+j} & =f_{3}^{A}+f_{4}^{B}+f_{1}^{C}+f_{2}^{D}\\ & \;-\left[\left[\begin{array}{ccc} l_{1} & l_{2} & l_{3}\\ l_{4} & l_{5} & l_{6}\\ l_{7} & l_{8} & l_{9} \end{array}\right]\right]c_{fr}\left(\left(i+x_{0}\right)h,jh\right),\\ & \;\;\;i=0,\ldots ,n,\;j=1,\ldots ,n. \end{array}$$

**Figure 5:**
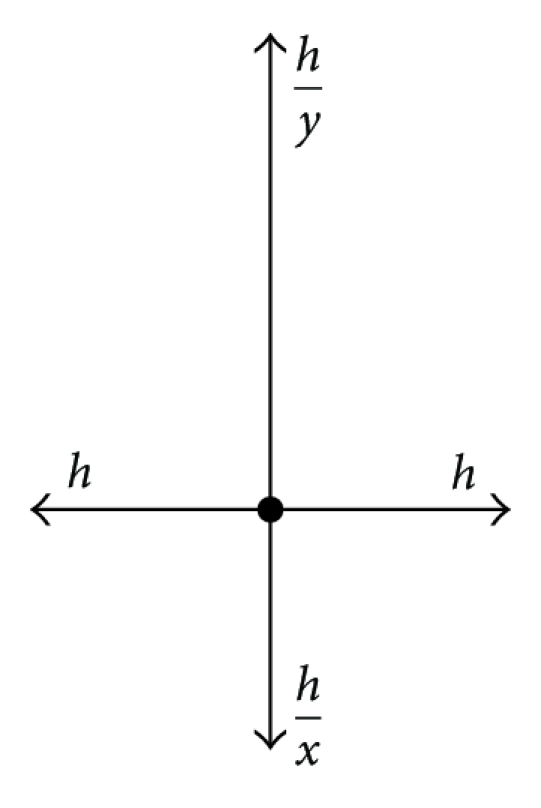


In the matrix *A*: *x* = *x*_0_ and *y* = *x*_0_ for the first line of blocks in (10), on *Ω*_*A*_ and *Ω*_*B*_, *x* = 1 and *y* = 1 on *Ω*_*C*_ and *Ω*_*D*_, while *x* = 1, *y* = 1 for the last line of blocks on *Ω*_*A*_ and *Ω*_*B*_, and *x* = 1 − *x*_0_, *y* = 1 − *x*_0_ on *Ω*_*C*_ and *Ω*_*D*_. For the remainder of the lines: *x* = 1, *y* = 1 (see Figure 5).

**Figure 6:**
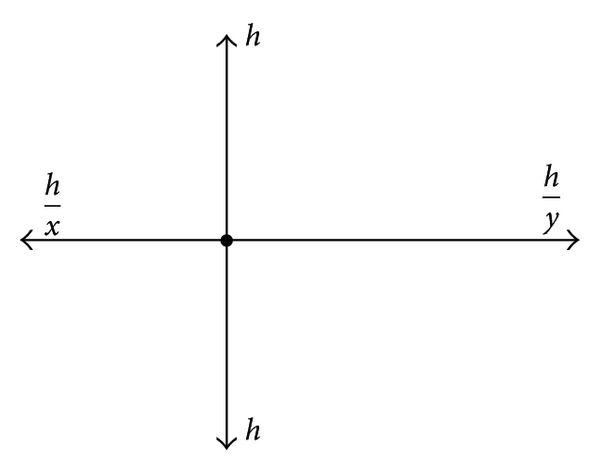


For the matrix *B*: *x* = 1/*x*_0_ and *y* = 1 for the first line of blocks on *Ω*_*A*_ and *Ω*_*D*_, *x* = 1 and *y* = 1 on *Ω*_*B*_ and *Ω*_*C*_. The last line has: *x* = 1, *y* = 1 on *Ω*_*A*_ and *Ω*_*D*_, and on *Ω*_*B*_ and *Ω*_*C*_: *x* = 1/(1 − *x*_0_), *y* = 1. For the other lines: *x* = 1, *y* = 1 (see Figure 6). 

**Figure 7:**
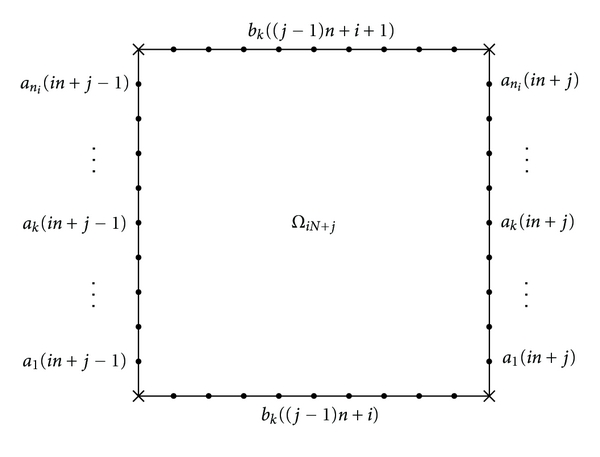


The system obtained by discretizing the problem on every subdomain *Ω*_*iN*+*j*_, *i* = 0,…, *n*, *j* = 1,…, *n* has *n*_*i*_^2^ equations and unknowns and will be solved using the Gauss full elimination method. The solutions *a* and *b* computed above will be used as boundary conditions on this domain (Figure 7).

Reuniting the solutions computed on the grid corresponding to the level *l*_0_ and the ones from every subdomain, one gets the final solution on the work level *l*.

## 5. Numerical Results and Conclusions

The method presented above allows the computing of the concentration of the solvent in blood at any point of the grid for different grid steps, and can be done for various dimensions of the vessel or values of the coefficients. The importance of such a method comes from the fact that the evaluation of cutaneous circulation can be a predictive parameter for the age-related pathology.

The values of the parameters used here are as follows:

- the diffusion coefficient: *m* = 10^−3^  cm^2^ s^−1^;
- the velocity vector: **n** = (10,10)  cm  s^−1^;
- the wall permeability: *ξ* = 1  cm  s^−1^;
- the concentration on Γ_in_: *g*_1_ = 2 · 10^−3^;
- the concentration on Γ_out_: *g*_1_ = 1.98 · 10^−3^;
- the forcing term for the solute concentration *f* = 0;
- the vessel dimensions have been chosen to be 1  cm × 0.2  cm.

**Figure 8:**
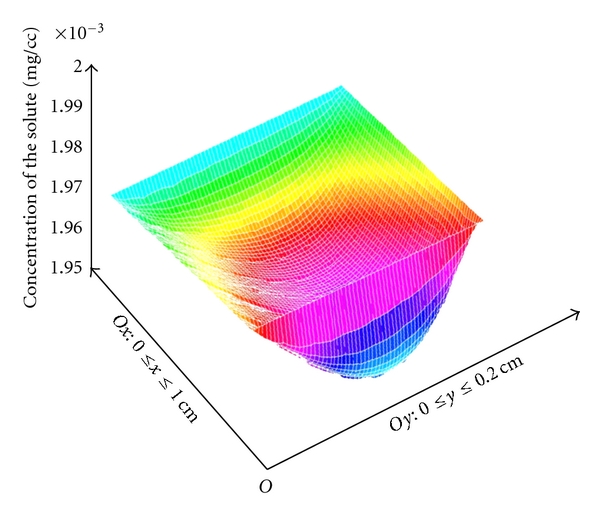
The concentration computed on the domain [0,1]×[0,0.2].

**Figure 9:**
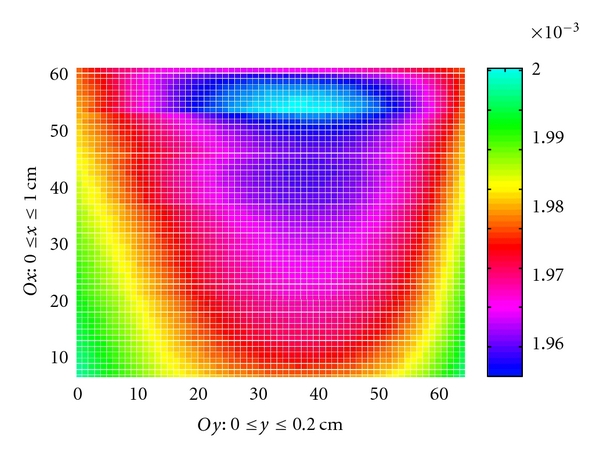
The concentration map on the domain [0,1]×[0,0.2].

For the convection-diffusion problem (1), the results are presented in Figures 8 and 9.
